# Detailed Protocol to Perform Direct PCR Using Filamentous Fungal Biomass—Tips and Considerations

**DOI:** 10.21769/BioProtoc.4889

**Published:** 2023-11-05

**Authors:** Hosung Jeon, Hokyoung Son, Kyunghun Min

**Affiliations:** 1 Department of Agricultural Biotechnology, Seoul National University, Seoul, Republic of Korea; 2 Research Institute of Agriculture and Life Sciences, Seoul National University, Seoul, Republic of Korea

**Keywords:** Direct PCR, Filamentous fungi, Molecular identification, ITS region

## Abstract

The precise and rapid detection of fungi is important in various fields, including clinics, industry, and agriculture. While sequencing universal DNA barcodes remains the standard method for species identification and phylogenetic analysis, a significant bottleneck has been the labor-intensive and time-consuming sample preparation for genomic DNA extraction. To address this, we developed a direct PCR method that bypasses the DNA extraction steps, facilitating efficient target DNA amplification. Instead of extracting genomic DNA from fungal mycelium, our method involves adding a small quantity of mycelium directly to the PCR mixture, followed by a heat shock and vortexing. We found these simple adjustments to be sufficient to lyse many filamentous fungal cells, enabling target DNA amplification. This paper presents a comprehensive protocol for executing direct PCR in filamentous fungi. Beyond species identification, this direct PCR approach holds promise for diverse applications, such as diagnostic PCR for genotype screening without fungal DNA extraction. We anticipate that direct PCR will expedite research on filamentous fungi and diagnosis of fungal diseases.

Key features

• Eliminates the time-consuming genomic DNA extraction step for PCR, enhancing the speed of molecular identification.

• Adds a small quantity of mycelium directly into the PCR mix.

• Emphasizes the crucial role of heat shock and vortexing in achieving efficient target DNA amplification.

• Accelerates the molecular identification of filamentous fungi and rapid diagnosis of fungal diseases.

**Graphical overview**


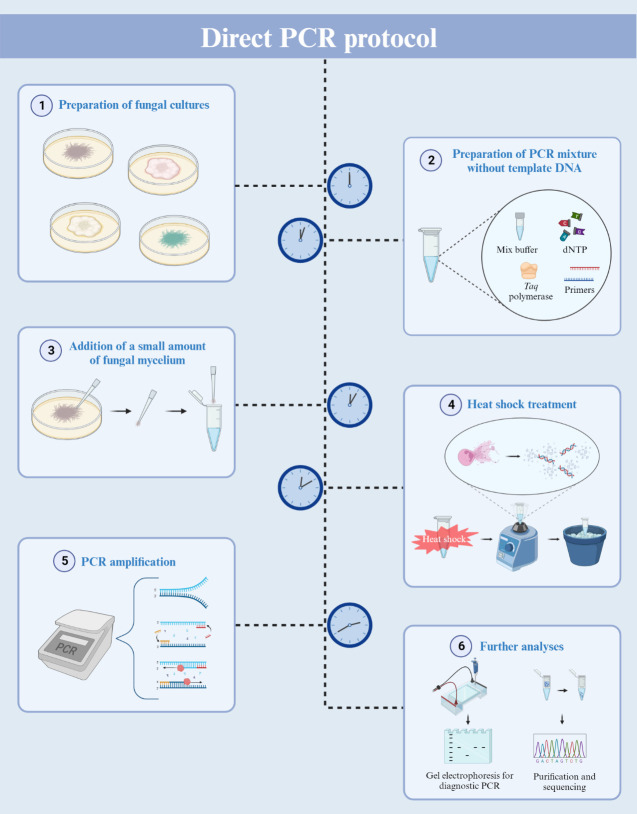


**Direct PCR using filamentous fungal biomass**

## Background

Polymerase chain reaction (PCR) is a powerful tool widely used to amplify specific target regions of DNA. Since its invention in the mid-1980s [1, 2], PCR has become a fundamental technique employed in various fields. A notable application of PCR is in the diagnosis of pathogenic diseases. It allows for the amplification of universal DNA barcodes, facilitating the identification of causative pathogens [3, 4]. Given that multiple copies of DNA are generated from the DNA template, the preparation of this template is essential for performing PCR. For filamentous fungi, the steps for DNA extraction and purification have been prerequisites for PCR amplification. Most fungi possess a rigid cell wall, predominantly constituted of polysaccharides like chitin and glucans [5, 6]. This results in inefficient fungal DNA extraction. The procedure for extracting DNA from fungal sources requires laborious and time-consuming steps involving the grinding of freeze-dried mycelia [7]. Additionally, the fungal DNA extraction process involves multiple stages using toxic chemicals like phenol and chloroform. A technique known as colony PCR involves directly adding cells into the PCR mixture, and it has found widespread application in bacterial and yeast research [8]. In the case of filamentous fungi, previous studies have suggested various PCR methods to minimize fungal DNA extraction steps. The methods proposed by Alshahni et al. and Walch et al. eliminated the need for fungal genomic DNA extraction [9, 10]. However, these methods incorporated additional steps, such as using lysis buffer or bovine serum albumin (BSA), to lyse fungal cell walls. Despite these advancements, the adoption of these methods for filamentous fungi has been limited due to their reduced PCR efficiency and the need for a cell lysis process.

Here, we introduce a direct PCR procedure tailored for filamentous fungi. This approach eliminates the need for the fungal DNA extraction process and the use of additional reagents such as BSA or proteinase. Our method involves adding a small amount of mycelium directly into the PCR mixture, followed by heat shock and vortexing. These heat shock and vortexing steps are key in breaking the fungal cell walls and membrane, releasing the genomic DNA for PCR amplification. This method will expedite the molecular identification of filamentous fungi and facilitate rapid diagnosis of fungal diseases.

## Materials and reagents

1. Potato dextrose broth (Difco, catalog number: 254920)
2. Agar powder (Duksan, catalog number: 601)
3. AccuPower® Taq PCR Premix (Bioneer, catalog number: 20-K-2602)
4. Nuclease-free water (Sigma-Aldrich, catalog number: 7732-18-5)
5. Primers:
  ITS4: 5′-TCCTCCGCTTATTGATATGC-3′
  ITS5: 5′- GGAAGTAAAAGTCGTAACAAGG-3′
  EF1T: 5′-ATGGGTAAGGAGGACAAGAC-3′
  EF2T: 5′-GGAAGTACCAGTGATCATGTT-3′
6. SeaKem® LE agarose (Lonza, catalog number: 50004)
7. RedSafe nucleic acid staining solution (iNtRON, catalog number: 21141)
8. Tris ultrapure (Duchefa Biochemie, catalog number: T1501.1000)
9. Ethylenediaminetetraacetic acid (EDTA) disodium salt dihydrate (Sigma-Aldrich, catalog number: E5134-50G)
10. Glacial acetic acid (Sigma-Aldrich, catalog number: PHR1748)
11. 6× Loading buffer (Takara, catalog number: 9156)
12. 100 bp Plus DNA ladder (Bioneer, catalog number: D-1035)
13. Ethanol EMSURE ACS, ISO, Reag. PH Eur (1 L) (Merck Millipore, catalog number: 1.0098.1011)

**Solutions**

1. Culture medium (PDA) (see Recipes)
2. 50× TAE buffer (1 L) (see Recipes)

**Recipes**

1. **Culture medium (PDA)**
  **Table:** 

Reagent	Final concentration	Quantity
Potato dextrose broth	24 g/L	24 g
Agar powder	10 g/L	10 g
H_2_O	n/a	up to 1 L
2. **50× TAE buffer (1 L)**
*Note: Add Tris ultrapure and EDTA disodium salt dihydrate to approximately 700 mL of H_2_O and stir until dissolved. Carefully add the acetic acid and adjust the volume to 1 L.
  **Table:** 

Reagent	Final concentration	Quantity
Tris ultrapure	2 M	242 g
Glacial acetic acid	1 M	57.1 mL
EDTA disodium salt dihydrate	50 mM	18.61 g
H_2_O	n/a	up to 1 L

**Laboratory supplies**

1. Pipette tips (1,000, 200, 10 μL), DNase, RNase, DNA, & endotoxin free (Neptune)
2. Petri dish (90 mm × 15 mm) (SPL, catalog number 10090)
3. 1.5 mL microtubes (Axygen, catalog number: MCT-15-C)
4. MEGAquick-spin^TM^ Plus Total Fragment DNA Purification kit (iNtRON, catalog number: 17290)

## Equipment

1. Pipettes (0.5–10 μL, 2–20 μL, 20–200 μL, 100–1,000 μL) (Eppendorf, model: Research Plus^®^)
2. Microcentrifuges mini (Labogene, catalog number: LZ-1312)
3. Vortex Genie 2 (Scientific Industries, catalog number: SI-0256)
4. MiniAmp^TM^ thermal cycler (Applied Biosystems, catalog number: A37834)
5. Gel tray L (Takara, catalog number: AD210)
6. PowerPac^TM^ basic power supply (Bio-Rad, catalog number: 1645050)
7. Gel imaging system MaXidoc G2 (DAIHAN Scientific, catalog number: DH.WGD00300)
8. NanoDrop 2000 (Thermo Scientific, catalog number: ND-2000)

## Procedure

- **Preparation of fungal culture**
  1. Transfer an agar plug containing fungal mycelia onto a PDA plate.
  2. Incubate the agar plate for five days at 25 °C.
- **Direct PCR amplification**
  1. For a 20 μL reaction volume, add 0.5 μL of each primer (20 μM) to AccuPower^®^ Taq PCR Premix tubes.
  2. To dissolve the vacuum-dried premixture, add 19 μL of nuclease-free water into the PCR tubes.
  3. Gently scratch the fungal culture using a pipette tip to collect a small amount of mycelium (Figure 1).
    **Figure 1.**
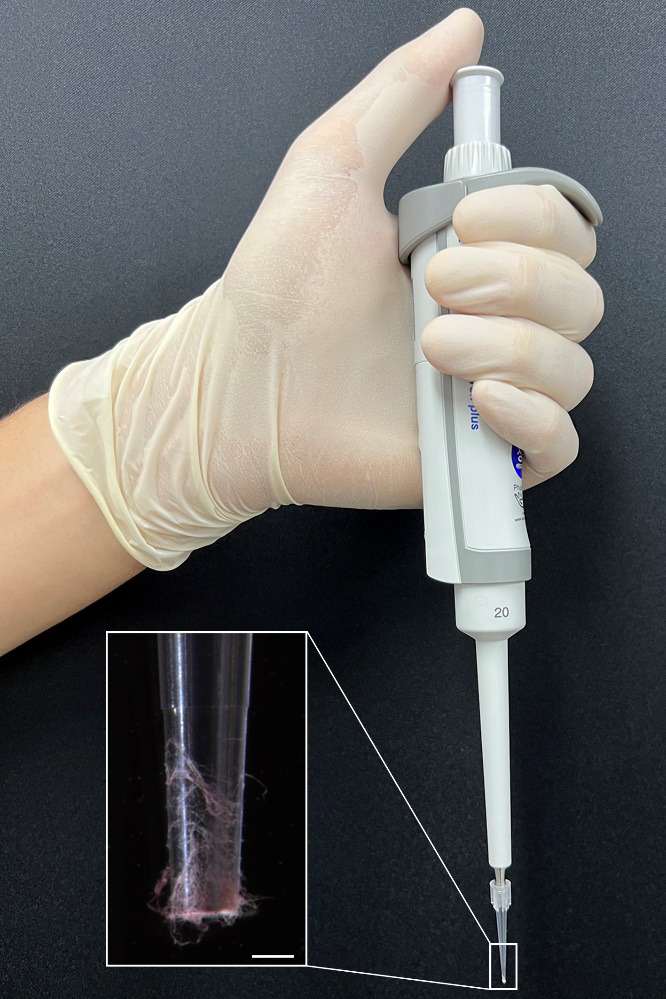
Mycelium collection for direct PCR. Aerial hyphae of *Fusarium graminearum* were collected using a pipette and 10 μL pipette tips. The distal part of the pipette tip is magnified for clarity. Scale bar = 500 μm.
  4. Transfer the collected mycelium to the reaction mixture. Vigorously vortex the PCR tubes and then briefly centrifuge to bring down the contents.
  5. For the heat shock reaction, place the PCR tubes in a thermal cycler and set the program (3 min at 95 °C, then cool down to 25 °C).
  6. After the program, immediately transfer the PCR tubes to an ice bath for 1 min.
  7. Vortex the samples vigorously for 15 s and then chill them on ice for an additional 15 s.
  8. Repeat step B7 two more times.
  9. Centrifuge the reaction mixture briefly to collect all contents at the bottom and then return the PCR tubes to the thermal cycler.
  10. Set the thermal cycler with the following program:
    - Initial denaturation at 95 °C for 1 min.
    - Denaturation at 95 °C for 30 s.
    - Annealing at 56 °C (adjust temperature based on primer specifications) for 1 min.
    - Extension at 72 °C (adjust time based on the expected amplicon size) for 1 min.
    - Repeat steps b–d for 40 cycles (based on our results, 40 cycles were more optimal than 30).
    - Final extension at 72 °C for 3 min.
    - Hold at 4 °C for 5 min.
- **Gel electrophoresis**
  1. Gel preparation:
    - Weigh 2.25 g of agarose powder.
    - Dissolve agarose powder in 150 mL of 1× TAE buffer (see Recipes) completely using a microwave (avoid overboiling) to make a 1.5 % agarose gel (w/v).
    - Add 7.5 μL of RedSafe nucleic acid staining solution and mix thoroughly.
    - Pour the dissolved agarose solution into a gel tray with a well comb.
    - Allow the poured gel to solidify fully at room temperature for 20–30 min.
  2. Loading samples and running:
  - Add 6× loading buffer to each PCR product.
  - Place the solidified agarose gel into the electrophoresis unit and submerge it entirely in 1× TAE buffer.
  - Carefully load your samples and 100 bp Plus DNA ladder into the gel wells.
  - Operate PowerPac^TM^ basic power supply at 100–120 V for 30–40 min (adjust the voltage and running time depending on gel concentration and PCR product size).
  - After gel running, carefully remove the gel from the unit and place it under a UV light device to visualize DNA fragments.
- **Purification and sequencing**
  1. To purify the DNA samples, use MEGAquick-spin^TM^ Plus Total Fragment DNA Purification kit and follow the protocol provided with the kit.
  2. Transfer 50 μL of your PCR product into a 1.5 mL microcentrifuge tube and add 250 μL of agarose gel lysis buffer (included in the DNA purification kit). Mix well by vortexing.
  3. Insert a spin column in a collection tube.
  4. Transfer the sample mixture to the spin column and centrifuge at 11,000× *g* for 30 s.
  5. Discard the flowthrough.
  6. Add 750 μL of the ethanol-added wash buffer solution (included in the kit) to the spin column and then centrifuge at 11,000× *g* for 30 s.
  7. Discard the flowthrough.
  8. Centrifuge again at 13,500× *g* for 3 min to dry the column matrix.
  9. Place the spin column in a new 1.5 mL microcentrifuge tube.
  10. Add 20–40 μL of elution buffer (included in the kit) to the membrane center of the spin column and wait for 3 min.
  11. Centrifuge at 13,500× *g* for 1 min to elute the DNA.
  12. Measure DNA concentration using a NanoDrop 2000 and submit the samples to a sequencing service center.

## Data analysis

DNA sequences were used to conduct a BLAST search against the NCBI GenBank database for species identification.

This protocol or parts of it has been used and validated in the following research article:

- Jeon et al. (2023) [4]. Application of direct PCR for phylogenetic analysis of *Fusarium fujikuroi* species complex isolated from rice seeds. Frontiers in Plant Science (Figures 1, 2, and 3).

## General notes and troubleshooting

1. The amount of mycelium added can have a significant effect on the PCR outcome. Always ensure that only a small quantity of mycelium is added to the PCR mixture. Introducing too much mycelium may lead to reaction blockages and potential failure (Figure 2).
  **Figure 2.**
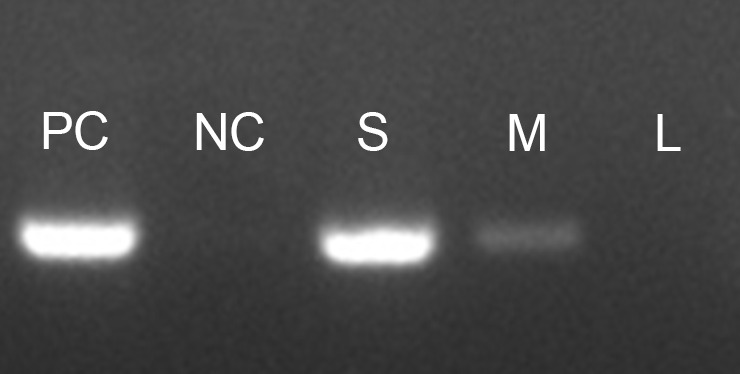
Influence of mycelium quantity on direct PCR efficiency. *F. graminearum* strain Z-3639 was subjected to amplification of the *TEF-1α* region using a direct PCR approach. The different quantities of mycelium affected PCR efficiency as follows: PC: positive control (using extracted genomic DNA), NC: negative control (no template control), S: small amount of mycelium (as shown in Figure 1), M: medium amount (twice that of S), and L: large amount (twice that of M).
2. While direct PCR can be performed using various PCR enzymes, we strongly recommend using the Taq PCR Premix. This premix contains all necessary components for the reaction (including polymerase, dNTP, reaction buffer, and loading dye), except for the template DNA and primers. Our observations show that Taq PCR Premix offers a notably higher success rate compared to other PCR enzymes. Furthermore, the presence of a loading dye in the Taq PCR Premix improves visualization of the added mycelium, allowing for easier verification of mycelium quantity.
3. In the previous study [4], direct PCR amplification of the ITS region was tested on samples from major fungal lineages including Ascomycota, Basidiomycota, and Mucoromycota. Our method displayed a remarkable PCR success rate of 92% (34 out of 37 fungal species). Nonetheless, the ITS region of three species—*Mucor mucedo, Aspergillus niger*, and *Aspergillus brasiliensis*—remained unamplified by direct PCR. Notably, these fungi exhibited highly hydrophobic mycelium and spores, which likely inhibited cell lysis within the PCR mixture.
4. In addition to the ITS, the *TEF-1α* region and other genotype markers were successfully amplified using the direct PCR method to identify strains within the *Fusarium fujikuroi* species complex.
5. If the PCR efficiency is low, consider increasing the duration of vortexing after the heat shock step.
6. It is advisable to include a reaction containing isolated genomic DNA as a positive control in each experiment. Also, always have a no-template control. This approach can provide valuable insights when troubleshooting is necessary.
